# Cancer mortality attributable to cigarette smoking in 2005, 2010 and 2015 in Qingdao, China

**DOI:** 10.1371/journal.pone.0204221

**Published:** 2018-09-20

**Authors:** Zhenshi Xu, Fei Qi, Yani Wang, Xiaorong Jia, Peng Lin, Meiyun Geng, Rui Wang, Shanpeng Li

**Affiliations:** 1 Medical College, Qingdao University, Qingdao, Shandong Province, China; 2 Qingdao Municipal Center for Disease Control and Prevention, Qingdao, Shandong Province, China; National Health Research Institutes, TAIWAN

## Abstract

**Introduction:**

Cigarette smoking is among the most important public health concerns worldwide and the leading preventable cause of illness and death associated with cancer, chronic obstructive pulmonary disease (COPD) and cardiovascular disease (CVD). Although Qingdao, China implemented smoking control measures in 2007 and smoke-free legislation in 2013, smoking-attributable cancer mortality remains at a high level. The present study aimed to facilitate changes in policy-making, intervention implementation, monitoring and evaluation by estimating and comparing the burden of smoking-attributable cancers in Qingdao during 2005, 2010 and 2015.

**Methods:**

This study used the disease list from the Global Burden of Disease (GBD) study to quantify the burden of smoking-related cancer. Sex and age-specific smoking-attributable mortality data were collected from the Qingdao Municipal Center for Disease Control and Prevention using an online reporting system. The population-attributable fractions (PAFs) of smoking and smoking-attributable cancer mortality in 2005, 2010 and 2015 were estimated using the smoking impact ratio (SIR) and relative risks (RRs) and by multiplying the smoking-attributable fraction by total cancer mortality, respectively.

**Results:**

The numbers of smoking-attributable cancer deaths increased from 2484 in 2005 to 2999 in 2010 and 4148 in 2015, with corresponding PAFs of 26.41%, 25.76% and 29.13%, respectively. The PAFs were higher among men (vs. women) for all cancers except cervical cancer. In 2005, lung, liver, esophageal and stomach cancers were most frequently associated with smoking-associated cancer mortality, and lung cancer had the greatest PAF, followed by nasopharyngeal, oral and esophageal cancers. Similar patterns were observed in 2010 and 2015. In 2015, 1 in 3 and 1 in 5 cancer deaths in men and women, respectively, were attributable to smoking, and 95% of these deaths were associated with lung, liver, esophageal or stomach cancer. Over time, downward and upward trends in smoking-attributable cancer deaths were respectively observed among people younger than and older than 50 years.

**Conclusions:**

The smoking-attributable cancer burden in Qingdao remains considerable, despite the implementation of tobacco control and smoke-free measures. Tobacco control efforts should remain a major public health priority.

## Introduction

Worldwide, tobacco smoking is regarded as one of the riskiest health behaviors and the leading factor responsible for various chronic non-communicable diseases, including cardiovascular diseases (CVD), respiratory diseases and cancers of the lungs and other organs[1]. In 2000, approximately 4.8 million adult deaths worldwide were attributed to smoking[2], and this number was projected to increase to 5.4 million in 2005, 6.4 million in 2015 and 8.3 million in 2030[3].

Currently, China has the largest tobacco production and consumption rates worldwide[4] and a current smoking rate of 27.7% among residents older than 15 years of age (52.1% for men, 2.7% for women); additionally, an estimated 3.16 hundred million residents are current smokers[5]. Smoking-related diseases are consistently listed among the top causes of death, in China; here, 1 million residents died from tobacco-related illnesses in 2010, and this number is expected to reach 2 million by 2030[6]. Findings from a national study suggest that tobacco smoking is responsible for 32.7% of all cancer deaths in Chinese men and 5% of cancer deaths in Chinese women[7]. Despite the health consequences, smoking remains highly prevalent in China, and tobacco control measures have been ineffective. The average age at which Chinese residents begin smoking has decreased from 22 years in 1984 to 19 years in 1996 and 18 years in 2002, despite the implementation of the Law of the People’s Republic of China on the Protection of Minors (<18 years) in 1999, which prohibited the sale of cigarettes to minors. These facts indicate that the existing national programs and initiatives regarding smoking prevention and cessation must be strengthened if cancer mortality is to be reduced in China[8].

Qingdao, a developed city in eastern China, is facing an aging population, which is an important risk factor for cancer. In 2005, the total population was 7,409,311, of which the population older than 65 years accounted for 10.5%; however, by 2010, the total population had increased to 7,636,392, of which the population over 65 years accounted for 12.1%. The total population increased further to 7,831,075, of which the population over 65 years accounted for 13.4% in 2015. Tobacco smoking is also the leading cause of cancer in Qingdao. Although Qingdao first implemented tobacco control measures in 2007 and subsequent smoke-free legislation in 2013, a 2014 study of the disease burden attributable to cigarette smoking in this city indicated a further increase in smoking-attributable cancer mortality[9]. Given the enormous burdens imposed by smoking on health, up-to-date information and analyses of the related patterns of change are important. Therefore, this study aimed to estimate the population-attributable fractions (PAFs) of smoking and smoking-attributable cancer mortality in Qingdao in 2005, 2010 and 2015.

## Methods

### Data sources

Based on the 2013 Global Burden of Disease (GBD) Study, 12 major smoking-related cancers were included in this study. The relative risks (RRs) of target cancers among smokers were also obtained from the 2013 GBD[10]. The RRs for the 12 major smoking-related cancers and ICD-10 (International Classification of Diseases, 10th Revision) codes are given in Table 1.

**Table 1:** Relative risks and diagnostic codes for 12 major smoking-related cancers.

ICD-10 Codes	Diseases	Men	Women
C15	Esophageal cancer	6.676	6.357
C16	Stomach cancer	1.927	1.57
C22	Liver cancer	2.540	1.724
C33,C34	Lung cancer	22.511	14.095
C18-C21	Colorectal cancer	1.325	1.418
C00-C08	Oral Cancer	8.162	6.056
C11	Nasopharynx cancer	8.227	6.089
C25	Pancreatic cancer	2.506	2.098
C64	Kidney cancer	2.293	1.518
C67	Bladder Cancer	3.332	2.582
C91-C95	Leukemia	2.013	1.163
C53	Cervical cancer		1.679

Population data were obtained from the Qingdao Municipal Bureau of Statistics. The scope of this analysis was limited to adults older than 30 years, as most cumulative hazardous effects of smoking-related cancers are unlikely to manifest in individuals younger than middle age. Stratification of the population by sex and age is shown in Table 2.

**Table 2:** The population of Qingdao by sex and age in 2005, 2010 and 2015.

	2005			2010			2015		
Age group (years)	Men	Women	Total	Men	Women	Total	Men	Women	Total
30–34	396208	393625	789833	266119	274832	540951	281054	297600	578654
35–39	391473	388720	780193	322301	323013	645314	268517	278502	547019
40–44	274286	266622	540908	370668	376136	746804	322243	324618	646861
45–49	342419	328020	670439	355434	353960	709394	367385	376141	743526
50–54	244176	236549	480725	285066	279927	564993	348112	352206	700318
55–59	176907	161416	338323	291377	288074	579451	274812	276819	551631
60–64	145083	130230	275313	189725	185748	375473	278951	284106	563057
65–69	134970	135714	270684	133486	126877	260363	177752	180978	358730
70–74	103312	117623	220935	117495	115500	232995	118423	119125	237548
75–79	64475	83806	148281	89944	104590	194534	95975	101975	197950
80–84	34552	50736	85288	52220	73609	125829	63720	82484	146204
85+	17487	34020	51507	27700	51051	78751	38729	68602	107331
Total	2325348	2327081	4652429	2501535	2553317	5054852	2635673	2743156	5378829

Disease-specific mortality data were obtained from the Qingdao Municipal Center for Disease Control and Prevention using an online reporting system associated with the sample vital registration (VR) system, which is administered by the National Health and Family Planning Commission (NHFPC) of the People’s Republic of China. This system covered the entire population of registered Qingdao households in 10 districts. Medical institutions at all levels used this system to collect death certificates and report related information to the Qingdao Municipal Center for Disease Control and Prevention. For deaths occurring at medical institutions, death certificates were completed by physicians according to standard protocols. For deaths occurring at home, death certificates were completed by local community physicians based on the descriptions of symptoms and personal histories of illness provided family members. The death certificates were coded according to the 10th revision of the International Classification of Diseases (ICD-10). The missing report rate was approximately 5%.

Lung cancer mortality data of the reference population were obtained from the American Cancer Society (ACS) Cancer Prevention Study Phase II (CPS II) and used to calculate the smoking impact ratio (SIR)[11]. The CPS-II was a prospective cohort study of smoking and death in a cohort of more than 1 million Americans older than 30 years. The majority of the exposed group in that study were life-long smokers, with a mean consumption of approximately 20 cigarettes per day who were considered representative of accumulated smoking exposure.

The study was approved by the Institutional Review Board of the Qingdao Municipal Center for Disease Control and Prevention. All data used in this study were fully anonymized before accession.

### Calculation of the population-attributable fraction (PAF) and smoking attributable mortality (SAM)

As the current prevalence of smoking is a poor proxy for the cumulative hazards of smoking, Peto et al.[11] proposed substituting the SIR for the smoking prevalence in the attributable fraction formula for cancers, as this would allow for the long lag between exposure and outcome. The SIR can be calculated as follows:

$$SIR=\frac{C_{LC}-N_{LC}}{S_{LC}^{*}-N_{LC}^{*}}$$

where C_LC_ is the lung cancer mortality rate in the study population, N_LC_ is the lung cancer mortality rate of never-smokers in the same population and S*_LC_ and N*_LC_ are the lung cancer mortality rates for smokers and never-smokers, respectively, in a reference population. Because local data were unavailable, the Chinese never-smoker lung cancer death rates were substituted for the non-smoker lung cancer mortality death rates in Qingdao[6].

The rate of lung cancer among non-smokers is much higher among the Chinese population than among the CPS-II population, as individuals in the former are exposed to other lung cancer risk factors, such as coal used for heating and cooking[12]. Therefore, the previous formula was normalized as follows[13]:

$$SIR=\frac{C_{LC}-N_{LC}}{S_{LC}^{*}-N_{LC}^{*}}\times \frac{N_{LC}^{*}}{N_{LC}}$$

The fraction of deaths attributable to smoking was estimated using the standard population-attributable fraction (PAF). The accumulated hazards of smoking in the Qingdao population were measured using the SIR which was used to calculate PAFs. The sex- and age-specific SIR and relative risk (RR) were substituted into the PAF formula as follows:

$$\text{PAF}=\frac{SIR(RR-1)}{SIR(RR-1)+1}$$

The formula used to determine the smoking-attributable mortality (SAM) was as follows:

SAM=PAR×M

where M denotes the sex- and age-specific deaths associated with the 12 smoking-attributable cancers.

## Results

As shown in Table 3, tobacco smoking was responsible for 2484, 2999 and 4148 cancer-related deaths in 2005, 2010 and 2015, respectively. Although upward trends in smoking-attributable mortality were observed over time for both men and women, the numbers of smoking-attributable deaths were higher among men. Notably, the approximately twofold increase in smoking-attributable mortality from 2010 to 2015 relative to that from 2005 to 2010 was largely due to an increase in deaths among men.

**Table 3:** Numbers of cancer deaths attributable to smoking and population-attributable fractions (PAFs) of diseases by sex in 2005, 2010 and 2015.

Diseases	Deaths attributable to smoking									PAF%								
	2005			2010			2015			2005			2010			2015		
	Men	Women	Total	Men	Women	Total	Men	Women	Total	Men	Women	Total	Men	Women	Total	Men	Women	Total
Esophageal cancer	177	22	199	201	28	229	272	21	293	33.62	23.47	32.08	31.88	24.16	30.67	36.25	25.13	35.14
Stomach cancer	112	29	140	118	28	146	154	31	185	8.09	4.28	6.85	7.19	3.73	6.12	8.41	3.78	6.98
Liver cancer	243	32	275	240	32	273	300	38	339	15.02	5.07	12.24	12.58	4.68	10.48	14.31	4.82	11.70
Lung cancer	1245	497	1741	1575	635	2210	2314	808	3122	63.99	45.11	57.16	62.20	44.38	55.77	66.84	46.50	60.04
Colorectal cancer	9	8	16	13	10	22	21	13	35	2.76	3.27	2.98	2.52	2.78	2.62	3.00	2.72	2.89
Oral Cancer	11	2	14	12	4	16	19	7	26	39.47	22.86	35.54	38.48	21.66	32.65	42.70	23.70	35.41
Nasopharyngeal cancer	21	4	25	19	2	22	21	5	26	43.74	25.72	38.96	39.16	35.43	38.69	42.57	26.63	37.95
Pancreatic cancer	20	8	28	27	9	36	40	15	55	12.59	7.11	10.39	11.42	6.59	9.62	13.21	7.01	10.62
Kidney cancer	6	1	7	5	1	7	9	2	10	11.49	3.92	9.36	9.00	3.95	7.32	10.73	3.21	7.82
Bladder Cancer	17	3	19	16	3	18	28	4	32	14.64	8.18	13.23	13.52	7.97	12.32	16.67	8.73	14.93
Leukemia	13	3	16	14	2	16	17	2	18	10.95	2.23	6.39	8.22	1.63	5.37	8.97	1.28	5.76
Cervical cancer		4			5			8			8.03			5.99			5.43	5.42
Total	1873	612	2484	2241	758	2999	3195	953	4148	29.69	19.73	26.41	28.40	20.20	25.76	33.00	20.92	29.13

Table 3 stratifies smoking-attributable cancer mortality by cancer type. In 2005, smoking-attributable mortality was most frequently attributable to lung cancer, followed by liver, esophageal and stomach cancers. Similar patterns were observed in 2010 and 2015. The four leading types of cancer associated mortality deaths (lung, liver, esophageal and stomach cancers) accounted for approximately 95% of the total cancer mortality attributable to smoking in all three study years. Furthermore, the proportion of lung cancer deaths attributable to smoking—the cancer with the highest PAF—increased over time from 57.16% in 2005 to 55.77% in 2010 and 60.04% in 2015, while the proportion of esophageal cancer deaths attributable to smoking also increased from 32.08% in 2005 to 30.67% in 2010 and 35.14% in 2015. Meanwhile, no changes were observed in the proportions of deaths associated with the other top-two smoking-attributable cancers, which were 38.96% in 2005, 38.69% in 2010 and 37.95% in 2015 for nasopharyngeal cancer and 35.54% in 2005, 32.65% in 2010 and 35.41% in 2015 for oral cancer.

Table 3 also stratifies the PAFs of diseases by sex. In 2005, the PAF for mortality associated with the 12 major cancers was 26.41%, with rates of 29.69% for men and 19.73% for women. These rates increased to 25.76%, or 28.40% for men and 20.20% for women, in 2010 and 29.13%, or 33.00% for men and 20.92% for women, in 2015.

**Fig 1:**
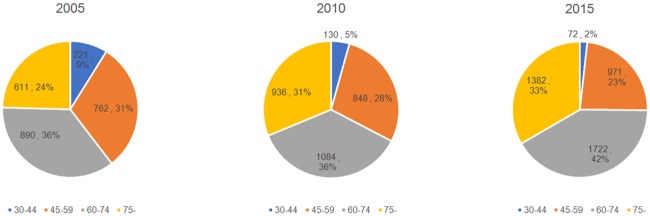
Ratios of smoking-attributable cancer deaths by age in Qingdao in 2005, 2010 and 2015.

Table 4 summarizes the numbers of cancer deaths attributable to smoking by age in 2005, 2010 and 2015. The PAF of people younger than 45 years decreased over time, from 37.21% in 2005 to 22.89% in 2010 and 17.84% in 2010. From 2005 to 2015, smoking-related mortality decreased among subjects younger than 50 years but increased gradually among those older than 55 years. As shown in Fig 1, the contribution of the younger population (30–44 and 45–59 years of age) to all smoking-attributable cancer deaths decreased gradually from 2005 to 2015, while the contribution of the older population (60–74 and >75 years) increased.

**Table 4:** Number of deaths attributable to smoking by age and sex in 2005, 2010 and 2015.

Age group (Years)	Deaths attributable to smoking									PAF%								
	2005			2010			2015			2005			2010			2015		
	Men	Women	Total	Men	Women	Total	Men	Women	Total	Men	Women	Total	Men	Women	Total	Men	Women	Total
30–34	15	10	25	7	5	12	3	5	8	34.00	33.23	33.70	21.47	23.53	22.31	14.18	19.39	16.84
35–39	46	24	70	21	12	33	10	8	18	36.85	42.76	38.71	20.97	22.45	21.49	15.04	22.13	17.42
40–44	88	38	126	50	35	85	24	23	46	37.88	35.63	37.17	19.09	35.30	23.57	14.28	25.64	18.20
45–49	165	28	193	159	37	196	136	18	154	39.89	16.96	33.28	37.16	21.29	32.60	34.35	10.39	27.10
50–54	245	57	302	204	54	258	229	62	291	37.99	25.22	34.65	30.64	22.87	28.62	31.01	22.76	28.79
55–59	208	59	267	320	74	394	439	87	526	33.18	24.57	30.78	32.27	21.45	29.46	38.73	22.75	34.71
60–64	229	40	269	244	68	312	523	115	637	31.34	17.51	28.04	26.39	20.65	24.88	36.82	22.52	33.04
65–69	205	63	269	259	75	334	457	110	567	24.63	16.93	22.24	28.76	20.08	26.21	36.20	20.77	31.65
70–74	250	103	353	334	105	439	382	136	518	24.41	19.55	22.76	28.29	20.12	25.79	31.27	25.23	29.43
75–79	227	89	316	301	112	413	406	130	536	27.53	17.13	23.51	25.91	18.36	23.31	29.97	19.21	26.38
80–84	128	61	189	221	101	323	352	131	483	24.40	17.17	21.47	28.17	17.85	23.84	30.76	18.63	26.15
85+	68	37	106	122	79	201	233	131	363	23.68	14.19	19.16	26.33	18.88	22.79	31.05	20.75	26.35
Total	1873	612	2484	2241	758	2999	3195	953	4148	29.69	19.73	26.41	28.40	20.20	25.76	33.00	20.92	29.13

## Discussion

In this study, we estimated the burden of smoking-attributable cancer mortality, a very important issue in Qingdao, and determined that these cancers accounted for 26.41% (29.69% for men, 19.73% for women), 25.76% (28.40% for men, 20.20% for women) and 29.13% (33.00% for men, 20.92% for women) of all cancer deaths in 2005, 2010 and 2015 respectively. In brief, the PAF in Qingdao is slightly higher than the national rate (30.6% for men, 6.2% for women)[14], and the cancer burden attributable to smoking has become a serious issue. This study demonstrates that the number of deaths associated with smoking-attributable cancers increased from 2484 in 2005 to 2999 in 2010 and 4148 in 2015, and this increasing trend was particularly strong among participants older than 55 years.

The 2014 Global Adult Tobacco Survey (GATS), conducted in Qingdao, observed the highest smoking rate among participants aged 45–69 years[15], which is considered a high-risk age range for smoking-related diseases[16]. This group tends to have deep-rooted smoking habits and therefore is resistant to tobacco control legislation and other interventions intended to promote smoking cessation. Furthermore, China began transitioning to an aging society in approximately 2000, and the proportion of the population aged older than 60 years is expected increase to 20.77% in 2025[17]. Generally, the increase in smoking-related deaths among people older than 55 years is attributed to the overlapping effects of a high smoking rate and population aging. Therefore, health resources should target the early diagnosis and proper treatment of smoking-related cancers in older populations.

International tobacco control experiences have demonstrated that the implementation of a range of effective tobacco control measures can reduce the smoking rate by 1% per year[18]. To address the above-described substantial health and economic burdens associated with smoking-attributable cancers, in 2007 the Qingdao government began to implement a series of tobacco control measures aimed at reducing the smoking rate. These measures included banning smoking in workplaces, protecting children from environmental tobacco smoke and banning tobacco advertising, sponsorships and promotions. In addition, Qingdao enacted a law that would make virtually all enclosed public places and workplaces smoke-free on September 1, 2013. These measures have successfully reduced the smoking rate in Qingdao to a lower level in recent years, as demonstrated by a 2014 survey[15] in which the current smoking rate among Qingdao residents aged at least 15 years, 21.31% (40.53% for men, 1.03% for women), was found to be lower than the current national smoking rate[5]. Furthermore, a survey of smoking rates in urban areas of China[19] found that Qingdao had the lowest smoking rate among the 14 surveyed cities.

In this study, decreases in smoking-attributable cancer deaths were observed over time in the younger population (<50 years) and particularly among those aged 30–44 years; in this age group, the proportion of smoking-attributable cancer-related deaths decreased from 9% to 2% from 2005 to 2015. A nationwide prospective cohort study predicted that men born during the 1970s and 1980s will likely be the first generation of men to experience the full hazards of tobacco smoking, as they reached adulthood when the nationwide cigarette consumption level was high[6] (average daily cigarette consumption per adult man in China: 1 in 1952, 4 in 1972, and 10 in 1992[20]). In other words, we are likely experiencing the early stage of effective measures against the threat of tobacco, which is expected to present an immense public health challenge. Furthermore, although smoking-attributable cancer mortality was low among the younger population in this study, additional strategies to counter the effects of tobacco will be needed to reduce cases of disability and premature death over the next few decades.

Like other cities in China, the smoking rate among men is much higher than that among women in Qingdao[15]. Accordingly, we observed a much higher overall increase in smoking-attributable cancer deaths among men, as well as higher rates of smoking-attributable cancer deaths and PAFs among men for all 12 established smoking-attributable cancers (other than cervical cancer) at all three time points. However, among some younger age groups (<45 years), female subjects had higher PAFs, although this was attributed to the use of the 40–44-year age group to estimate the SIR for age groups younger 40 years because this was the only age group with a higher proportion of women relative to men at all three study time points. A previous study of the burden of tobacco-related cancer in China[14] observed an overall PAF of approximately 6.2% for female subjects, which was far below the level observed in Qingdao. This discrepancy could be attributed to the high rate of lung cancer in Qingdao. A previous epidemiological study observed a high incidence of lung cancer mortality in Qingdao, with an associated death rate far higher than the national average and the average rates of major cities in China[21]. In 2010, the age-standardized lung cancer mortality rates among Qingdao and Chinese women according to the Chinese standard population were 21.75/10^5^ and 16.62/10^5^, respectively; in other words, the lung cancer mortality rate among women is higher in Qingdao than in all of China. In addition, it is worth noting that women are susceptible to the harmful effects of second-hand smoking. In a previous study conducted in China, approximately 11% of lung cancer deaths among non-smoking women were attributable to exposure to smoke from a spouse or in the workplace[7]. Despite the reduced smoking rate in Qingdao, secondhand smoke exposure rates remained high in 2014, with rates of 42.39%, 26.62% and 40.53% reported in public places, workplaces and homes, respectively[22]. We note that the ban on smoking in public places may have increased the exposure of women to secondhand smoke in their homes. Moreover, the smoking rate has begun to increase among women in China; particularly, the rate among young women is increasing by 10% annually[4]. Therefore, despite the relatively lower smoking-attributable cancer burden among women, proper tobacco control interventions should be implemented to discourage women from taking up the habit of smoking.

This study found that lung cancer was the leading cause of smoking-related cancer deaths in Qingdao, consistent with both national[8] and global research[23]. Although the PAFs were highest for lung, nasopharyngeal, oral and esophageal cancers, overall deaths were most frequently associated with lung, liver, esophageal and stomach cancers because of the low incidence of nasopharyngeal and oral cancers. In Qingdao, the number of deaths from lung cancer increased rapidly from 2005 to 2010 and 2015, which unfortunately confirms earlier predictions that the incidence of lung cancer would increase enormously consequent to the rapid increase in the number of cigarette smokers in China in the 1980s[24, 25]. Consistent with this prediction, the lung cancer incidence rate increased by an average of 1.63% each year (1.3% in men and 2.34% in women) from 1988 to 2005[26]. A Chinese study found that 98.54% of the lung cancer disability-adjusted life years (DALYs) are attributable to lung cancer years of life lost (YLLs), [27] as lung cancer is highly fatal (5-year survival rate of 19.8% in China[28]) and patients have a very short survival time (median time to death is 13.67 months in Beijing, China [29]).

These results underscore the need for government-based measures that reinforce tobacco control and advocate early diagnostic screening for smokers, with the aim of reducing lung cancer mortality by preventing deaths and/or providing effective treatment.

The lag time between the implementation of tobacco control measures (smokers quitting or young people never starting to smoke) and the decrease in smoking-related cancer mortality is very long because smoking-related cancers develop after approximately 20–30 years of continuous smoking, and the risk of cancer decreases slowly among former smokers. Therefore, it is important to implement and strengthen tobacco control measures as soon as possible. In November 2014, the Beijing People’s Congress passed the Beijing Smoking Control Regulation which is the most powerful tobacco control regulation in Chinese history. This regulation, which took effect on June 1, 2015, bans smoking in all indoor areas of public places, workplaces and public transport, as well as outdoor areas such as kindergartens, schools and child and maternal hospital campuses. Violators face a penalty of ¥50–200 (US$8–32) as an individual or ¥2000–10 000 (US$322–1611) as a venue owner or manager. The government also set up a complaint hotline to facilitate public reporting of smoking in public places. A study of the effectiveness of the Beijing Smoking Control Regulation found that the rate of observed smoking incidents in 176 participating restaurants decreased significantly from 40.3% to 14.8% (p<0.001) after the law came into force. Although Qingdao has also promulgated a tobacco control regulation, the implementation has not been sufficiently strict, and the stipulated fines imposed on violators are generally not implemented. The government of Qingdao should further strengthen the implementation of this regulation to reduce the overall disease burden caused by smoking, as this would help not only to reduce the burden of cancer, but also other of non-communicable diseases and some communicable diseases. One previous study found that a reduction in smoking of 30% from the 2013 level would lead to the avoidance of 326000 deaths due to noncommunicable diseases (e.g., cancer, cardiovascular diseases, diabetes mellitus and chronic respiratory diseases), including 222400cancer-related deaths, among 30–70-year-old Chinese residents by 2030[30].

Some limitations of this study should be noted. First, the RRs used in our analysis were not limited to the Chinese population. RRs based on published studies from a global population may not be fully applicable to the Qingdao population because of possible differences in smoking habits. Second, the Chinese never-smoker lung cancer death rate was used as a substitute for the non-smoker lung cancer mortality rate in the Qingdao population when calculating the SIR. Accordingly, this rate may not have accurately depicted the cumulative harm of smoking in Qingdao. Finally, this study did not account for former smokers and thus may have underestimated the burden of mortality attributable to smoking.

## Conclusion and suggestion

In conclusion, we observed a steady increase in the incidence of smoking-attributable cancer deaths from 2005 to 2010 and 2015. In 2015, 1 in 3 and 1 in 5 cancer deaths in men and women, respectively, were attributable to smoking, and 95% of these deaths were related to lung, liver, esophageal or stomach cancer. Furthermore, we note that despite a decrease in the number of smoking-attributable deaths among residents younger than 55 years, the burden of tobacco smoking-related cancer remains a serious issue among older residents and remains high among women, compared to the national level. We hope that our findings will provide public health experts and decision makers with a better understanding of the societal burden imposed by smoking on the Qingdao population. Although the government of Qingdao has already implemented successful public health and legislative tobacco control measures, our findings suggest that health policies targeted at specific populations are needed to further reduce the mortality attributable to smoking. These policies should prioritize awareness regarding the adverse effects of smoking in men, among whom the smoking prevalence is 40 times higher than among women. Furthermore, early screening, diagnosis and treatment measures are required for the older population, and awareness programs regarding the adverse effects of secondhand smoke should be developed to target women. Finally, it is essential to strengthen the implementation of tobacco control measures, such as the prohibition of tobacco advertisements and sales of cigarettes to minors, and to impose fines on violators of tobacco control regulations.
